# Supplementary material for: The Evolution and Characterization of the RNA Interference Pathways in Lophotrochozoa
Source: Genome Biol Evol. 2024 May 7;16(5):evae098. doi: 10.1093/gbe/evae098 (PMC11114477; doi:10.1093/gbe/evae098)
Supplement: evae098_Supplementary_Data [file evae098_supplementary_data.zip › Supplementary_materials.zip!/selected_miRNAs/NoGe_selected_miRNAs/mirdeep_pdf_files/NMRB01004296.1_Notospermus_geniculatus_strain_Ushimado_scaffold4296,_whole_genome_shotgun_sequence_45764.pdf]

Provisional ID	:	NMRB01004296.1_Notospermus_geniculatus_strain_Ushimado_scaffold4296,_whole_genome_shotgun_sequence_45764
Score total	:	6183.8
Score for star read(s)	:	3.9
Score for read counts	:	6175.1
Score for mfe	:	1.7
Score for randfold	:	0
Score for cons. seed	:	3
Total read count	:	12124
Mature read count	:	12114
Loop read count	:	0
Star read count	:	10

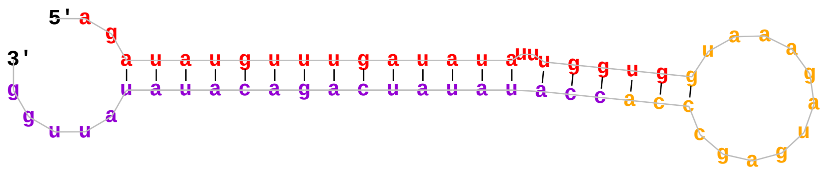

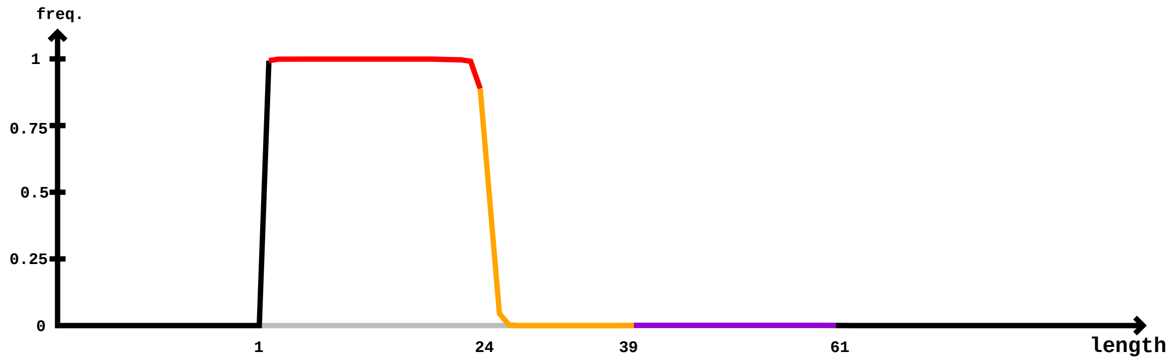

Mature   Star

```
5'- aaaucaaccucgucccuccuagauauguuugauauauuugguggguaaagaugagcccaccauauaucagacauauauuggaguggugagccgaccaacacuuaccgugguu  -3'   obs
    aaaucaaccucgucccuccuagauauguuugauauauuugguggguaaagaugagcccaccauauaucagacauauauuggaguggugagccgaccaacacuuaccgugguu         exp
    ........((((.((((((...((((((((((((((..((((((...........))))))))))))))))))))...)))).))))))..(((((.(.......))))))   reads   mm   sample
    ....................agauauguuugauauauuu........................................................................   1       0    Mm3
    ....................agauauguuugauauauuugg......................................................................   3       0    Mm3
    ....................agauauguuugauauauGuggu.....................................................................   1       1    Mm3
    ....................agauauguuugauauauuuggA.....................................................................   1       1    Mm3
    ....................agauauguuugauauauuuggu.....................................................................   78      0    Mm3
    ....................agauauguuuAauauauuuggu.....................................................................   1       1    Mm3
    ....................agauaCguuugauauauuuggug....................................................................   1       1    Mm3
    ....................agauauguuugauauauuuggug....................................................................   275     0    Mm3
    ....................agauauguuugauauauuugguU....................................................................   16      1    Mm3
    ....................agaGauguuugauauauuuggug....................................................................   1       1    Mm3
    ....................agauauNuuugauauauuuggug....................................................................   1       1    Mm3
    ....................agauauguuuAauauauuuggug....................................................................   2       1    Mm3
    ....................agauauguuugauauauuugguA....................................................................   7       1    Mm3
    ....................agauauguuugauauauAuggug....................................................................   1       1    Mm3
    ....................agauauguuugauauaCuuggug....................................................................   10      1    Mm3
    ....................agauauguuugauauaGuuggug....................................................................   9       1    Mm3
    ....................agauauguuugauauaCuuggugg...................................................................   1       1    Mm3
    ....................agauauguuugauauauuuggugU...................................................................   159     1    Mm3
    ....................agauauguuugauauauuuggugg...................................................................   32      0    Mm3
    ....................agauauguuugauauauAuggugg...................................................................   1       1    Mm3
    ....................agauauguuugauauauuuggugA...................................................................   4       1    Mm3
    ....................agauauguuugauauauuuggugUu..................................................................   2       1    Mm3
    ....................agauauguuugauauauuugguggA..................................................................   2       1    Mm3
    ....................agauauguuugauauauuugguggu..................................................................   4       0    Mm3
    ....................agauauguuugauauauuuggugguU.................................................................   1       1    Mm3
    .....................gauauguuugauauauuuggugU...................................................................   1       1    Mm3

    ....................agauauguuugauauauu.........................................................................   5       0    Fm3
    ....................agauauguuugauauauuA........................................................................   2       1    Fm3
    ....................agauauguuuAauauauuug.......................................................................   1       1    Fm3
    ....................agauauguuugauauauuug.......................................................................   5       0    Fm3
    ....................agauauguuugauauauuugg......................................................................   12      0    Fm3
    ....................aAauauguuugauauauuuggu.....................................................................   1       1    Fm3
```

```
                    Mature                              Star
aaaucaaccucgucccuccuagauauguuugauauauuuggugguaaagaugagcccaccauauaucagacauauauuggaguggugagccgaccaacacuuaccgugguu
....................agauauguuugauauaGuuggu.....................................................................    2     1     Fm3
....................agauauguuugauauaCuuggu.....................................................................    2     1     Fm3
....................agauauguuuUauauauuuggu.....................................................................    1     1     Fm3
....................agauauguuugauauauuuggA.....................................................................    3     1     Fm3
....................agauauguuugauauauAuggu.....................................................................    2     1     Fm3
....................Ggauauguuugauauauuuggu.....................................................................    1     1     Fm3
....................agauauguuugauGuauuuggu.....................................................................    2     1     Fm3
....................agauauguuuAauauauuuggu.....................................................................    1     1     Fm3
....................agauauguuugauauauuGggu.....................................................................    1     1     Fm3
....................agauauguuugauauauuuggu.....................................................................    113   0     Fm3
....................aUauauguuugauauauuuggug....................................................................    1     1     Fm3
....................agauauguuGgauauauuuggug....................................................................    1     1     Fm3
....................agCuauguuugauauauuuggug....................................................................    1     1     Fm3
....................agauauguuugauauauuuUgug....................................................................    1     1     Fm3
....................agauauguuugauauauGuggug....................................................................    6     1     Fm3
....................agauauAuuugauauauuuggug....................................................................    1     1     Fm3
....................Ugauauguuugauauauuuggug....................................................................    1     1     Fm3
....................agauauguuugauauauAuggug....................................................................    5     1     Fm3
....................agauauguuuAauauauuuggug....................................................................    2     1     Fm3
....................agauauNuuugauauauuuggug....................................................................    1     1     Fm3
....................agauauguuugauauauuugguU....................................................................    13    1     Fm3
....................Ngauauguuugauauauuuggug....................................................................    1     1     Fm3
....................agauauguuugauauauuugguA....................................................................    3     1     Fm3
....................agauGuguuugauauauuuggug....................................................................    1     1     Fm3
....................agauauguuugauaCauuuggug....................................................................    1     1     Fm3
....................agauauguuugauGuauuuggug....................................................................    6     1     Fm3
....................agauauguuugauauauuuggug....................................................................    999   0     Fm3
....................agauauguuugauauaGuuggug....................................................................    33    1     Fm3
....................agauauguuugauauauCuggug....................................................................    4     1     Fm3
....................agauauguuCgauauauuuggug....................................................................    1     1     Fm3
....................agauauguuugauauaCuuggug....................................................................    81    1     Fm3
....................agauauguuugauauauuuggAg....................................................................    1     1     Fm3
....................agGuauguuugauauauuuggug....................................................................    1     1     Fm3
....................agauauguuugauauauuuggugU...................................................................    483   1     Fm3
....................agauauguuugauGuauuuggugg...................................................................    1     1     Fm3
....................agauauguuugauauauuuggugg...................................................................    50    0     Fm3
....................agauauguuugauauauuuggugA...................................................................    6     1     Fm3
....................agauauguuugauauaCuuggugg...................................................................    1     1     Fm3
....................agauauguuugauauaGuuggugg...................................................................    1     1     Fm3
....................agauauguuugauauauuugguggu..................................................................    47    0     Fm3
....................agaGauguuugauauauuugguggu..................................................................    1     1     Fm3
....................agauauguuugauauauuuggugUu..................................................................    1     1     Fm3
....................agauauguuugauauauGugguggu..................................................................    1     1     Fm3
....................agauauguuugauauaCuugguggu..................................................................    1     1     Fm3
....................agauauguuugauauauuuggugggC.................................................................    1     1     Fm3
....................agauauguuugauauauuuggugguU.................................................................    5     1     Fm3
.....................gauauguuugauauauuuggu.....................................................................    3     0     Fm3
.....................gauauguuugauauauuuggug....................................................................    6     0     Fm3
.....................gauauguuugauauauuugguA....................................................................    1     1     Fm3
.....................gauauguuugauauauuuggugU...................................................................    3     1     Fm3
.....................gauauguuugauauauuugguggu..................................................................    1     0     Fm3
......................aGauguuugauauauuuggugg...................................................................    1     1     Fm3
..........................................................ccauauaucaCacauauauug................................    1     1     Fm3
..........................................................ccauauaucagacauauauug................................    2     0     Fm3

....................agauauguuugauauauuugg......................................................................    1     0     Mm1
....................agauauguuugauauauuuggu.....................................................................    22    0     Mm1
....................agauauguuugauauaCuuggug....................................................................    4     1     Mm1
....................agauauguuugauauaGuuggug....................................................................    3     1     Mm1
....................agauauguuugauauauuuggug....................................................................    85    0     Mm1
....................agauauguuugauauauGuggug....................................................................    1     1     Mm1
....................agauauguuugauauauuuggugA...................................................................    1     1     Mm1
....................agauauguuugauauauuuggugU...................................................................    11    1     Mm1
....................agauauguuugauauauuuggugg...................................................................    1     0     Mm1
....................agauauguuugauauauuugguggu..................................................................    3     0     Mm1
....................agauauguuugauauauuuggugUu..................................................................    3     1     Mm1

....................agauauguuugauauauuug.......................................................................    1     0     Mm2
....................agauauguuugauauauuugg......................................................................    9     0     Mm2
....................agauauguuugauauauAuggu.....................................................................    2     1     Mm2
```

```
                    Mature                 Star
aaaucaaccucgucccuccuagauauguuugauauauuuggugguaaagaugagcccaccauauaucagacauauauuggaguggugagccgaccaacacuuaccgugguu
....................agauauguuugauauauuuggu.....................................................................	89	0	Mm2
....................agauCuguuugauauauuuggu.....................................................................	1	1	Mm2
....................agauauguuugUuauauuuggug....................................................................	1	1	Mm2
....................agauauguuugauauauuuAgug....................................................................	1	1	Mm2
....................Ugauauguuugauauauuuggug....................................................................	2	1	Mm2
....................agauauguuugauauauGuggug....................................................................	4	1	Mm2
....................agauauguuugauauauCuggug....................................................................	2	1	Mm2
....................agauauguuugauauaCuuggug....................................................................	11	1	Mm2
....................agauauguuugauauauuuggug....................................................................	428	0	Mm2
....................agauauguuuAauauauuuggug....................................................................	1	1	Mm2
....................agauauguuugauauauuugguA....................................................................	1	1	Mm2
....................agauauguuugauauaGuuggug....................................................................	10	1	Mm2
....................agGuauguuugauauauuuggug....................................................................	1	1	Mm2
....................agaGauguuugauauauuuggug....................................................................	1	1	Mm2
....................agauauguuugauauauuugguU....................................................................	14	1	Mm2
....................agauauguuugauauauuuggugA...................................................................	7	1	Mm2
....................agauauguuugauauauuuggugg...................................................................	78	0	Mm2
....................agauauguuugauauauCuggugg...................................................................	1	1	Mm2
....................agauauguuugauauauuuggugU...................................................................	219	1	Mm2
....................agauauguuugauauaCuuggugg...................................................................	1	1	Mm2
....................agauauguuugauauauuugguggA..................................................................	1	1	Mm2
....................agauauguuugauauauuugguggG..................................................................	1	1	Mm2
....................agauauguuugauauauCugguggu..................................................................	1	1	Mm2
....................agauauguuugauauauuugguggu..................................................................	55	0	Mm2
....................agauauguuugauauauAugguggu..................................................................	1	1	Mm2
....................agauauguuugauauauuuggugUu..................................................................	2	1	Mm2
....................agauauguuugauauauuugguggguU.................................................................	5	1	Mm2
.....................gauauguuugauauauuuggug....................................................................	1	0	Mm2
.....................gauauguuugauauauuugguggu..................................................................	2	0	Mm2
.......................uauguuugauauauuuggug....................................................................	1	0	Mm2
........................auguuugauauauuuggu.....................................................................	1	0	Mm2
.........................................................accauauaucagacauauauug................................	1	0	Mm2

....................agauauguuugauauauuA........................................................................	3	1	Fm2
....................agauauguuugauauauuugg......................................................................	5	0	Fm2
....................agauauguuugauauauCugg......................................................................	1	1	Fm2
....................agauauguuugauauaGuuggu.....................................................................	1	1	Fm2
....................agauauguuugauauauCuggu.....................................................................	4	1	Fm2
....................agauauguuugauauauGuggu.....................................................................	1	1	Fm2
....................Ugauauguuugauauauuuggu.....................................................................	1	1	Fm2
....................agauauguuugauauaCuuggu.....................................................................	4	1	Fm2
....................agauauguuugauauauuuggu.....................................................................	324	0	Fm2
....................agauauguuugauauauuuggug....................................................................	2759	0	Fm2
....................agaCauguuugauauauuuggug....................................................................	1	1	Fm2
....................agUuauguuugauauauuuggug....................................................................	1	1	Fm2
....................agauauguuugGuauauuuggug....................................................................	1	1	Fm2
....................agauauguuugauUuauuuggug....................................................................	1	1	Fm2
....................agauauguuugauauauGuggug....................................................................	16	1	Fm2
....................agauauguuugauauaGuuggug....................................................................	82	1	Fm2
....................agauauguuGgauauauuuggug....................................................................	1	1	Fm2
....................agauauguuuAauauauuuggug....................................................................	1	1	Fm2
....................agGuauguuugauauauuuggug....................................................................	1	1	Fm2
....................agauauguuugauauaCuuggug....................................................................	185	1	Fm2
....................agauauguuugauauauCuggug....................................................................	18	1	Fm2
....................agauauguuuUauauauuuggug....................................................................	4	1	Fm2
....................aAauauguuugauauauuuggug....................................................................	1	1	Fm2
....................Ngauauguuugauauauuuggug....................................................................	1	1	Fm2
....................agauauguuugauGuauuuggug....................................................................	1	1	Fm2
....................agauauguuuCauauauuuggug....................................................................	1	1	Fm2
....................agauauguuugauauauAuggug....................................................................	24	1	Fm2
....................agauauguuugCuauauuuggug....................................................................	1	1	Fm2
....................agauauguuugUuauauuuggug....................................................................	1	1	Fm2
....................agauauguuugauauauuugguA....................................................................	63	1	Fm2
....................agauauguuugauauauuuggCg....................................................................	1	1	Fm2
....................agauauguuugauauauuugguU....................................................................	51	1	Fm2
....................Cgauauguuugauauauuuggugg...................................................................	1	1	Fm2
....................agauauguuuCauauauuuggugg...................................................................	1	1	Fm2
....................agauauguuugauauauuuggugU...................................................................	1131	1	Fm2
....................agauauguuuAauauauuuggugg...................................................................	1	1	Fm2
....................agauauguuugauauauuuggugg...................................................................	276	0	Fm2
```

Mature Star

```
aaaucaaccucgucccuccuagauauguuugauauauuuggugguaaagaugagcccaccauauaucagacauauauuggaguggugagccgaccaacacuuaccgugguu
....................agauauguuugauauauGuggugg...................................................................  2     1  Fm2
....................agauauguuugauauaCuuggugg...................................................................  6     1  Fm2
....................agauauguuugauauaCuugguggu..................................................................  1     1  Fm2
....................agauauguuugauauauuugguggC..................................................................  1     1  Fm2
....................agUuauguuugauauauuugguggu..................................................................  1     1  Fm2
....................agauauguuugauauaGuugguggu..................................................................  1     1  Fm2
....................agauauguuugauauauuuggugUu..................................................................  9     1  Fm2
....................agauauguuugauauauuugguggu..................................................................  119   0  Fm2
.....................gauauguuugauauauuuggug....................................................................  13    0  Fm2
.....................gauauguuugauauauuuggugC...................................................................  1     1  Fm2
.....................gauauguuugauauauuuggugg...................................................................  21    0  Fm2
.....................gauauguuugauauauGuggugg...................................................................  1     1  Fm2

....................agauauguuugauauauu.........................................................................  9     0  Fm1
....................agauauguuugauauauuu........................................................................  4     0  Fm1
....................agauauguuugauauauuug.......................................................................  2     0  Fm1
....................agauauguuugauauauuugg......................................................................  34    0  Fm1
....................agauauguuugauauauuuggA.....................................................................  4     1  Fm1
....................agauauguuugauauauCuggu.....................................................................  3     1  Fm1
....................agauauguuugCuauauuuggu.....................................................................  1     1  Fm1
....................agauauguCugauauauuuggu.....................................................................  1     1  Fm1
....................agauauguuugauauaGuuggu.....................................................................  1     1  Fm1
....................agauauguuugauauauuuUgu.....................................................................  1     1  Fm1
....................agGuauguuugauauauuuggu.....................................................................  1     1  Fm1
....................agauauguuugauauauAuggu.....................................................................  1     1  Fm1
....................agauauguuugauauaCuuggu.....................................................................  4     1  Fm1
....................agauauguuugauauauGuggu.....................................................................  2     1  Fm1
....................agauauguuugauauauuuggu.....................................................................  564   0  Fm1
....................Ggauauguuugauauauuuggu.....................................................................  1     1  Fm1
....................agauauguuugauauauuuggC.....................................................................  1     1  Fm1
....................agauauguuugauauauAuggug....................................................................  15    1  Fm1
....................agauauguuuCauauauuuggug....................................................................  1     1  Fm1
....................agauauguuuAauauauuuggug....................................................................  3     1  Fm1
....................agauauguuugauGuauuuggug....................................................................  2     1  Fm1
....................agauauguuugauauauuuggug....................................................................  1271  0  Fm1
....................agauGuguuugauauauuuggug....................................................................  1     1  Fm1
....................agGuauguuugauauauuuggug....................................................................  1     1  Fm1
....................agauauguuugauauaGuuggug....................................................................  12    1  Fm1
....................agaGauguuugauauauuuggug....................................................................  3     1  Fm1
....................agauaCguuugauauauuuggug....................................................................  1     1  Fm1
....................agauauguuugauauauuugguA....................................................................  41    1  Fm1
....................agauauguuugauauauuugUug....................................................................  1     1  Fm1
....................Ngauauguuugauauauuuggug....................................................................  2     1  Fm1
....................agauauguuugauauUuuuggug....................................................................  1     1  Fm1
....................agauauguuugauauauGuggug....................................................................  6     1  Fm1
....................agauauguuugauauauCuggug....................................................................  12    1  Fm1
....................agauauguuugauauauuugguU....................................................................  88    1  Fm1
....................Ugauauguuugauauauuuggug....................................................................  1     1  Fm1
....................agauauguuugauauaCuuggug....................................................................  25    1  Fm1
....................agauauguuugauauauuugguC....................................................................  3     1  Fm1
....................agauauguuugauauauuuggugC...................................................................  1     1  Fm1
....................agauauguuugauauauuuggugg...................................................................  171   0  Fm1
....................agauauguuugauauauuuggugU...................................................................  730   1  Fm1
....................agauauguuuAauauauuuggugg...................................................................  1     1  Fm1
....................agauauguuugauauauCuggugg...................................................................  1     1  Fm1
....................agauauguuugauauaCuuggugg...................................................................  2     1  Fm1
....................agauauguuugauauaGuuggugg...................................................................  1     1  Fm1
....................aNauauguuugauauauuuggugg...................................................................  1     1  Fm1
....................agauauguuugauauauGuggugg...................................................................  2     1  Fm1
....................agauauguuugauauauuuggugA...................................................................  15    1  Fm1
....................agauauguuugauauauuugguggG..................................................................  1     1  Fm1
....................agauauguuugauauCuuugguggu..................................................................  1     1  Fm1
....................agauauguuugauauauuugguggA..................................................................  4     1  Fm1
....................agauauguuugauauauuuggugUu..................................................................  15    1  Fm1
....................agauauguuugauauaCuugguggu..................................................................  4     1  Fm1
....................agauauguuugauauauuugguggu..................................................................  219   0  Fm1
....................agauauguuugauauauGugguggu..................................................................  1     1  Fm1
....................agauaugAuugauauauuugguggu..................................................................  1     1  Fm1
....................agauauguuugauauauAugguggu..................................................................  2     1  Fm1
....................agauauguuugauauauCugguggu..................................................................  2     1  Fm1
```

Mature Star

```
aaaucaaccucgucccuccuagauauguuugauauauuuggugguaaagaugagcccaccauauaucagacauauauuggaguggugagccgaccaacacuuaccgugguu
....................agauauguuugauGuauuuggugu...................................................................    1    1    Fm1
....................agauauguuugCuauauuuggugu...................................................................    1    1    Fm1
....................Ngauauguuugauauauuuggugu...................................................................    1    1    Fm1
....................agauauguuugauauauuugguguU..................................................................    14   1    Fm1
.....................gauauguuugauauauuuggug....................................................................    4    0    Fm1
.....................gauauguuugauauauuCggug....................................................................    1    1    Fm1
.....................gauauguuugauauauuuggugg...................................................................    5    0    Fm1
.....................gauauguuugauauauuuggugU...................................................................    2    1    Fm1
.....................gauauguuugauauauuGggugg...................................................................    1    1    Fm1
..........................................................ccauauaucagacauauauug................................    1    0    Fm1
..........................................................ccauauaucagacauauauugU...............................    5    1    Fm1
```
